# Nonlinear Optical Response of Tungsten Carbide Thin Film as Saturable Absorber at 1 μm and Its Application for Passively Q-Switched Nd:YAG Lasers

**DOI:** 10.3390/nano15080605

**Published:** 2025-04-15

**Authors:** Zhonglin Zhang, Liang Xie, Zhengwu Liu, Xu Wang, Jiang Wang, Guodong Zhang, Xinwei Zhang, Zongcheng Miao, Guanghua Cheng

**Affiliations:** 1 School of Artificial Intelligence, Optics and Electronics (iOPEN), Northwestern Polytechnical University, Xi’an 710072, China; zhangzhonglin@mail.nwpu.edu.cn (Z.Z.); xielianggd@mail.nwpu.edu.cn (L.X.); zhengwu@mail.nwpu.edu.cn (Z.L.); miaozongcheng@nwpu.edu.cn (Z.M.); guanghuacheng@nwpu.edu.cn (G.C.); 2 School of Science, Xi’an Shiyou University, Xi’an 710065, China; 220201@xsyu.edu.cn; 3 School of Electronics and Information, Northwestern Polytechnical University, Xi’an 710119, China; zhangers79@163.com

**Keywords:** tungsten carbide, Q-switched solid-state laser, saturable absorber, thin film

## Abstract

Pulsed lasers have a wide range of applications in scientific and industrial fields, and the saturable absorber (SA) is the core device of pulsed lasers. Tungsten carbide (WC) has garnered significant attention due to its exceptional physicochemical properties, making it a promising candidate for optoelectronic applications, particularly as an SA in pulse lasers. This study is the first to report the nonlinear optical properties of WC thin film at a 1064 nm wavelength and its use as an SA device to generate pulsed lasers. A high damage threshold of 472.4 mJ/cm^2^ was achieved, which is a critical parameter for high-power laser applications. The constructed laser demonstrated pulsed output with a central wavelength of 1064.12 nm, an average output power of 185 mW, and a narrow pulse width of 684 ns. Our research has provided a strong candidate for the development of future economically stable high-power laser systems.

## 1. Introduction

Pulsed lasers, compared to continuous wave lasers, have the advantages of high peak power, narrow pulses, and high energy, and have been widely used in military, medical, industrial, and scientific research fields [1,2]. The saturable absorber (SA) is the core component of a pulsed laser, and the quest for an advanced SA has driven significant progress in pulsed laser technology, with materials like graphene and MXene leading the way in exploring new horizons of nonlinear optics and ultrafast electronics [3,4,5,6,7]. Despite the many advantages of these new SAs, they still face some challenges in practical applications, such as stability, reliability, fabrication costs, and integration difficulties. Therefore, the search for and development of new SA devices that are stable and cost-effective remain highly significant.

Tungsten carbide (WC), an outstanding member of the transition metal carbides, is widely recognized in the field of materials science due to its exceptional physicochemical properties. This extremely hard material boasts a high melting point of approximately 2870 °C and demonstrates remarkable chemical stability at room temperature, making it highly reliable and safe for storage and application [8]. The characteristics of WC have led to its widespread use in various fields, including chemical catalysis [9,10], battery electrodes [11], and material processing [12]. More notably, the unique electronic structure and excellent optical properties of WC suggest a promising future and substantial potential in the field of optoelectronic devices. However, despite its significant potential in many areas, research on WC as an SA is relatively scarce [13]. In 2023, Wang et al. demonstrated the potential of WC film-based SAs in ultrafast fiber lasers, highlighting their application in pulse lasers with central wavelengths of 1560 nm and 1932 nm [14]. But, the study of high-energy pulsed lasers in the 1064 nm band is still insufficient.

In this study, WC films were successfully deposited on a quartz glass substrate using magnetron sputtering technology. Further investigation using the Z-scan technique revealed the nonlinear optical characteristics of the WC films at 1.0 μm for the first time, with a saturation intensity and modulation depth of 7.595 GW/cm^2^ and 8.85%, respectively. After precise measurement, the damage threshold of the WC film SAs’ irradiation exceeded 472.4 mJ/cm^2^. Ultimately, by integrating the WC film SAs into the resonant cavity of an Nd:YAG laser, a 1064.12 nm passive Q-switched solid-state pulse laser was constructed, achieving an average output power of 185 mW and a narrow pulse width of 684 ns. This work demonstrates that WC is a promising material for saturable absorbers, which has significant reference value for the exploration of pulse laser materials.

## 2. Experimental Methods

The magnetron sputtering technique, known for its low deposition temperature, fast deposition speed, and good film uniformity, is highly suitable for industrial production and large-scale applications [15,16]. In this study, radio frequency magnetron sputtering technology was utilized to prepare WC films on quartz substrates. The target material used was WC with a purity of 99.99%, and the deposition substrate was quartz glass. The vacuum inside the sputtering chamber was achieved using a molecular pump, with the base vacuum level required to be below 4 × 10^−4^ Pa. Argon gas was selected as the sputtering gas, with a flow rate controlled at 30 sccm. By adjusting the gate valve between the introduction chamber and the sputtering chamber molecular pump, the introduction chamber and sputtering chamber were brought to a working pressure of 1 Pa, respectively.

**Figure 1:**
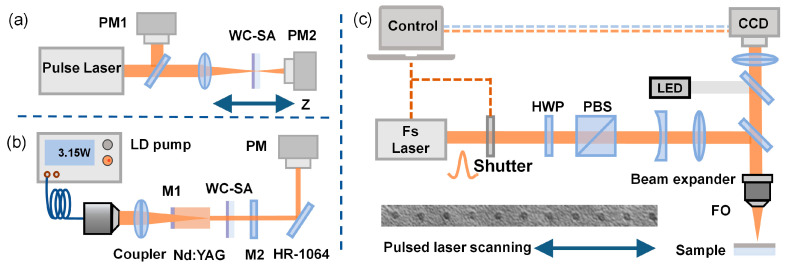
(**a**) Schematic of Z-scan twin-detector technique setup; (**b**) schematic diagram of the linear cavity of the Nd:YAG laser; and (**c**) experimental setup of the damage threshold test.

The open-aperture Z-scan measurement technique, utilizing ultrafast lasers, was employed to measure the third-order nonlinear optical absorption characteristics of WC materials. Figure 1a shows a schematic diagram of the open-aperture Z-scan experimental setup. An ultrafast laser, featuring a central wavelength of 1030 nm, a pulse width of 1.2 ps, and a repetition rate of 10 kHz, was employed as the light source. A beam splitter was used to equally divide the ultrafast pulse light into two parts with the same power, one serving as the reference light, and the other directed onto the WC film SA device through a lens. The beam waist radius at the focus was 19.4 μm.

In this experiment, an experimental device for a Nd:YAG solid-state laser was set up, as depicted in Figure 1b. The device employed a Nd:YAG crystal doped with 1.2% Nd^3+^ as the laser gain medium and used a commercial 808 nm continuous wave diode laser as the pumping source to enhance the pumping power and absorption efficiency. To ensure effective heat dissipation, the crystal was fixed on a copper block, which was connected to a circulating water-cooling system maintained at 17 °C. The coupling mirror M1 of the resonant cavity was formed by applying an 808 nm antireflection (AR) coating and a 1064 nm high-reflection (HR) coating to the front surface of the crystal. The output mirror M2 was a concave mirror, and its transmission rate for the 1064 nm wavelength was precisely set to 10%. To regulate the intracavity loss, the WC film SA was integrated into the laser cavity, facilitating the implementation of passive Q-switching and consequently yielding a stable pulsed output.

The laser-induced damage threshold is a critical parameter for evaluating the performance of SA optical modulators [17]. To accurately gauge the laser damage resistance of WC films, a specialized and precise testing system was constructed, with its layout elaborated in Figure 1c. The system consists of a stable laser source and a precision translation stage. The ultrafast laser system operates with a pulse duration of 1.2 ps and a repetition rate of 100 kHz at a wavelength of 1030 nm. By focusing the laser through a lens, a precise spot with a diameter of about 13.4 μm was formed on the WC film SA and each energy level corresponded to a row position on the sample. Further observation of the SA device using a white light interferometer allowed us to accurately capture the contour of the damaged area. As the laser energy density was gradually reduced, the damage to the SA device also decreased. Upon reaching a certain critical laser energy density, the damage to the WC film SA ceased to be observable, which allowed for the determination of the laser damage threshold range for the WC film SA [18].

## 3. Results and Discussions

**Figure 2:**
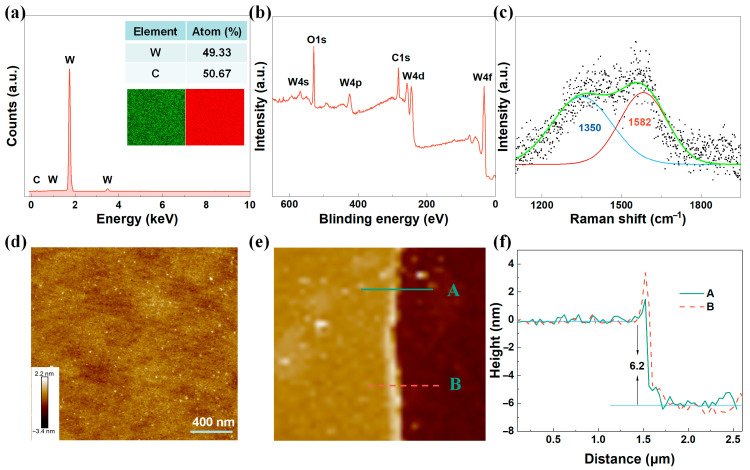
Characterization of the WC films: (**a**) EDS image, (**b**) XPS survey spectra, (**c**) Raman spectroscopy, (**d**) AFM image, and (**e**,**f**) cross-sectional height profile of the WC thin films.

In this study, an Oxford cold-field energy spectrometer (EDS, Oxford instruments, Oxford, UK) was employed for precise measurement. The EDS analysis results, as shown in Figure 2a, accurately reflected the elemental composition of the film, with C and W atoms accounting for 49.33% and 50.67%, respectively, precisely corresponding to the theoretical stoichiometric ratio of the WC compound. Moreover, no characteristic peaks of impurities were observed in the EDS spectrum, confirming the high chemical purity of the WC film. Furthermore, the element distribution map, shown in the inset of Figure 2a, revealed the uniform distribution of W and C elements in the film.

This study used X-ray photoelectron spectroscopy (XPS, AXIS ULTRA, Kyoto, Japan) technology for meticulous elemental analysis. Figure 2b shows the XPS measurement spectrum recorded over a wide energy range of 0 to 700 eV, from which the presence of W, C, and O can be clearly identified, directly confirming the chemical composition of the WC film [19,20]. In particular, the detection of O element (O 1s) can be attributed to the moisture absorbed on the surface or the possible presence of an oxidized tungsten layer, which is a common phenomenon in the preparation and processing processes [21]. The Raman spectrum shown in Figure 2c was obtained using a Raman spectrometer (Renishaw inVia Reflex, Gloucestershire, UK) at an excitation wavelength of 514 nm. The spectrum exhibits two significant Raman main peaks at 1350 cm^−1^ and 1582 cm^−1^. These peaks correspond to the D peak and G peak in carbon materials, which are key features in the Raman spectrum of carbon materials [22,23].

The thickness and surface roughness of the film are two crucial parameters for evaluating the quality of the film material. To accurately characterize these characteristics, an atomic force microscope (AFM, Bruker Dimension ICON, Berlin, Germany) was used for meticulous analysis. The surface morphology of the prepared WC film is visually presented through the AFM image, as shown in Figure 2d. The WC film surface roughness Rq value revealed by the AFM image was 0.22 nm, indicating that the WC film deposited via magnetron sputtering technology has extremely high smoothness and uniformity, which is beneficial for minimizing surface defects and thus improving the overall performance of the material. In addition, the film thickness data obtained through AFM technology, as shown in the AFM contour diagram in Figure 2e,f, determined that the thickness of the WC film was approximately 6.2 nm.

**Figure 3:**
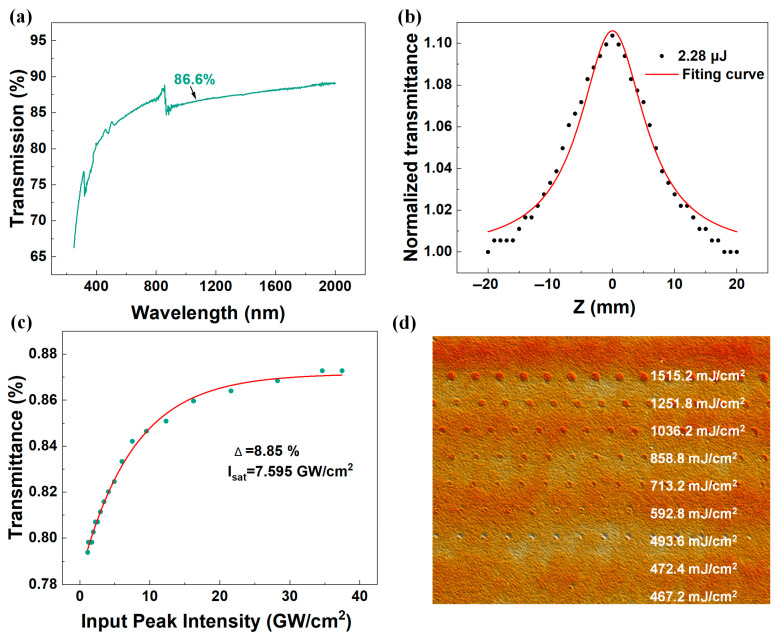
(**a**) Transmission spectra, (**b**) Z-scan, (**c**) relationship between normalized transmittance and input laser intensity, and (**d**) damage under different energy density conditions.

A PerkinElmer LAMBDA 1050^+^ UV/Vis/NIR spectrophotometer (PerkinElmer, Waltham, MA, USA) was used to conduct a detailed analysis of the transmission spectrum over the wavelength range of 200–2000 nm. As shown in Figure 3a, the transmittance of the WC film measured at 1064 nm reached 86.6%. In this study, an in-depth investigation of the saturable absorption characteristics of the WC film SA was conducted using the open-aperture Z-scan technique and a balanced synchronous dual-detector measurement method. The normalized transmittance T(z) measured using the Z-scan technique can be represented by Equation [24,25,26]:

(1) Tz=1−βI0Leff22(1+z2z02)

where *β* represents the nonlinear absorption coefficient, *I*_0_ represents the axial peak intensity at the focal point (*z* = 0), and *L_eff_* and *z*_0_ represent the effective length and Rayleigh range, respectively.

The corresponding data are shown in Figure 3b. By observing the fitting curve, it was noted that the normalized transmittance changes with the movement of the sample film, especially when approaching the optical axis focus (*z* = 0), where there is a significant increase in transmittance, a typical manifestation of the saturable absorption effect. The calculated nonlinear absorption coefficient of the WC film SA was −1.98 × 10^−4^ m/W. To further analyze the saturable absorption parameters of the WC film SA optical modulator, the experimental data were fitted using the following Equation [27,28,29]:

(2) $$T\left(I\right)=1-T_{ns}-\Delta \times exp\left(-\frac{I}{I_{sat}}\right)$$

where *T*(*I*) and Δ represent the transmittance and modulation depth, respectively. *I* and *I_sat_* correspond to the incident light intensity and saturation intensity, and *T_ns_* is the non-saturated absorbance. The experimental fitting results are shown in Figure 3c, with a high saturation intensity of 7.595 GW/cm^2^ and a calculated modulation depth of 8.85%.

A white light interferometer is an advanced non-contact three-dimensional profiling device that uses the principle of white light interferometry to perform high-precision scanning of the sample surface. In this study, the three-dimensional morphology of the ultrafast laser-induced damage area was successfully captured using a white light interferometer (Bruker ContourX-200, Berlin, Germany), as shown in Figure 3d. The experimental results show that as the laser energy density decreases, the degree of damage to the SA also decreases accordingly. Through precise measurement, the damage threshold range of the WC film SA was determined to be between 493.6 mJ/cm^2^ and 472.4 mJ/cm^2^ under specific laser parameters.

**Figure 4:**
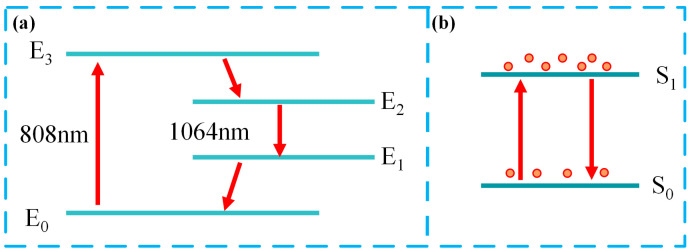
Energy level diagram of (**a**) Nd: YAG and (**b**) saturated absorber.

In our work, a WC film SA was integrated into the designed solid-state laser resonator cavity. By adjusting the pump power and optical modulator of the SA, the experiment achieved short-pulse laser outputs. The kinetics of passively Q-switched lasers are shown in Figure 4. Particles at the ground state energy level (E_0_) absorb pump energy and transition to the excited energy level (E_3_), followed by rapid relaxation at the upper laser level (E_2_). Subsequently, particles at the E_2_ level emit 1 μm photons and transition to the lower laser level (E_1_), before finally relaxing back to the ground state (E_0_).

The saturable absorber can be considered a two-level system, as shown in Figure 4b. When pumping is initiated, the saturable absorber strongly absorbs 1 μm photons and leads to strong intracavity losses. As the number of particles that transition to the excited state S_1_ energy level increases, the number of particles in the ground state S_0_ gradually decreases, and the absorption of 1 μm by the saturated absorber gradually weakens. Until the ground state particles of the saturated absorber are exhausted, the Q value of the laser resonant cavity reaches its maximum and emits laser light in the form of pulses. After laser emitting, the intracavity loss is once again greater than the gain, and the resonant cavity returns to the photon accumulation phase and repeats the cycle of the process.

**Figure 5:**
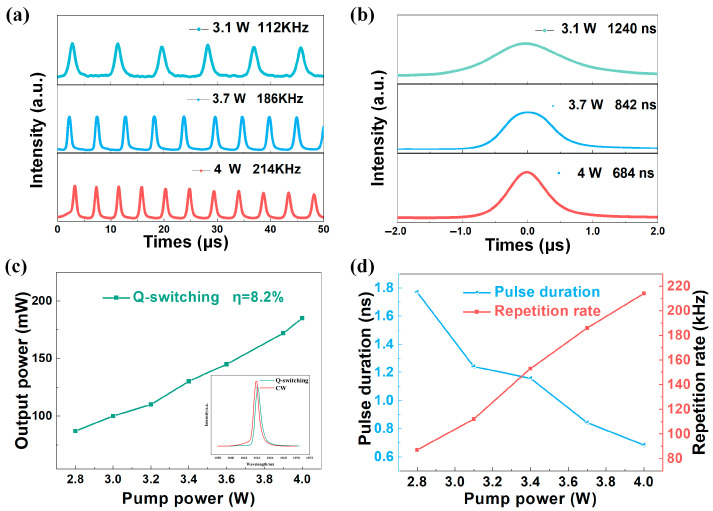
Q-switched operation: (**a**) typical laser pulse trains, (**b**) typical laser single pulses (**c**) average output power of Q-switched and emission spectrum, and (**d**) evolution of the repetition rate and pulse duration at the pump power.

As shown in Figure 5a, when the pump power is set to 3.1 W, 3.7 W, and 4.0 W, the output pulse frequencies are 112 kHz, 186 kHz, and 214 kHz, respectively. The corresponding pulse widths are 1240 ns, 842 ns, and 684 ns, as shown in Figure 5b. Furthermore, Figure 5c elucidates the dependence of average output power on pump power, which increases as the pump power increases. The insets in Figure 5c show the spectra under Q-switching (with the saturable absorber) and CW (without the saturable absorber), respectively. For the Q-switched operation, the center wavelength is 1064.12 nm, and the full width at half maximum (FWHM) is 0.98 nm. For the CW operation, the center wavelength is 1063.76 nm, and the FWHM is 0.90 nm. With an increase in pump power from 2.8 W to 4 W, a significant enhancement in average output power, from 87 mW to 185 mW, was observed, with a calculated optical conversion efficiency of about 8.2%. Additionally, the repetition frequency of the Q-switched solid-state laser changes with the increase in pump power. Figure 5d demonstrates the trend of pulse width and repetition frequency as the pump power is increased from 2.8 W to 4 W. The frequency expanded from 87 kHz to 214 kHz, and the pulse width correspondingly decreased from 1.77 μs to 0.684 μs. This trend reveals the operational mode of the laser at different pump powers.

To compare the nonlinear optical absorption properties and the output performance of pulsed solid-state lasers with other 2D material SAs, previous research results at the same wavelength of 1 μm are summarized in Table 1. Compared to other 2D materials as saturable absorbers (SAs), WC has successfully demonstrated pulse operation and exhibits an exceptionally high saturation intensity, as shown in Table 1. The high saturation intensity allows for maintaining higher light intensity within the resonant cavity when using WC as a saturable absorber, which is beneficial for achieving a narrow pulse output. Further work will be conducted in the future to explore this potential in greater detail.

## 4. Conclusions

In conclusion, the research presented in this paper has successfully demonstrated the fabrication and application of WC thin films as SAs for solid-state lasers through the magnetron sputtering technique. The WC films, when deposited on a quartz glass substrate, showcased remarkable material and optical properties, including a high modulation depth of 8.85% and a significant nonlinear optical response. The laser damage threshold testing further confirmed the robustness of the WC film, with a damage threshold exceeding 472.4 mJ/cm^2^, highlighting its suitability for high-power laser operations. The integration of the WC film into a passively Q-switched Nd:YAG laser cavity resulted in the generation of stable laser pulses at 1064 nm, with an average output power of 185 mW and a narrow pulse width of 684 ns. This study underscores the WC film’s potential as a novel and promising broadband SA for use in stable Q-switched pulse lasers.

## Figures and Tables

**Table 1:** Pulsed Q-switched lasers based on 2D saturable absorber.

2D Material	Laser Type	ΔT (%)	I_SA_	Repetition Rate (kHz)	Pulse Duration (ns)	Average Output Power (mW)	Ref.
Ti_3_C_2_Tx	Nd:YAG	36.7	1.07 μJ/cm^2^	186	359	94.8	[30]
ZrC	Nd:YAG	6.9	48.4 MW/cm^2^	596	78	121	[7]
ReS_2_	Nd:YAG	5.2	21.5 μJ/cm^2^	644	139	120	[31]
V_2_O_5_	Nd:YAG	21.8	329.8 kW/cm^2^	379	59	86	[32]
Cs_0.15_FA_0.85_PbI_2.85_Br_0.15_	Nd:YAG	15.1	6.14 MW/cm^2^	381.2	166	338	[33]
MoS_2_/SiO_2_	Nd:YAG	8.41	9.35 MW/cm^2^	1809	132	-	[34]
WC	Nd:YAG	8.85	7.595 GW/cm^2^	214	684	185	Our work

## Data Availability

The original contributions presented in this study are included in the article. Further inquiries can be directed to the corresponding author(s).

## References

[1] Wu Z., Wu X., Zhang Y., Liu Y., Zhang X., Yang C. (2024). Effect of nanosecond pulse laser power on welding interface and mechanical properties of AZ31B Mg/6061 Al. Opt. Laser Technol..

[2] Berczeli M., Tajti F., Juhász G., Weltsch Z. (2024). Changing the High Strength Steel Surface Properties with Femtosecond Laser Beam. Opt. Laser Technol..

[3] Zhang J., Sun R., Ge Y., Wang J., Wang Z., Meng L., Deepak F.L., Zhang M., Yin P., Cheng F. (2024). Atomic-scale structure and nonlinear optical absorption of two-dimensional GeS. J. Mater. Sci. Technol..

[4] Yang Z., Yang Q., Tian Y., Ren X., Li C., Zu Y., Din S.Z.U., Gao L., Wu J., Chen H. (2023). Few-layer Ti_3_CN MXene for ultrafast photonics applications in visible band. J. Mater..

[5] Chen Q., Lu S., Zhang Y., Yin H., Li Z., Zhang P., Chen Z. (2024). The passive Q-switched and Q-switched mode-locked Nd: GYAP laser based on a novel Bi_2_O_3_ saturable absorber. Opt. Laser Technol..

[6] Wang F., Qu Y., Lan D., Zhang X., Cheng T. (2022). VO_2_ nanoparticles saturable absorbers onto D-shaped fiber for mode-locked operation at 1560 nm band. Opt. Laser Technol..

[7] Wang J., Li G., Liu S., Chai J., Wang Y., Cheng G., Zhang G., Liu Y., Li X. (2023). Nonlinear absorption response of Zirconium Carbide films. ACS Appl. Mater. Interfaces.

[8] Polini R., Marcucci A., D’Ottavi C., Nunziante P., De Filippis P., Marcheselli G. (2021). Toward greener synthesis of WC powders for cemented tungsten carbides manufacturing. ACS Sustain. Chem. Eng..

[9] Rafique M., Fu Q., Han J., Wang R., Yao T., Wang X., Song B. (2024). Tungsten Carbide-Based Materials for Electrocatalytic Water Splitting: A Review. ChemElectroChem.

[10] Li H., Wang W., Xue S., He J., Liu C., Gao G., Di S., Wang S., Wang J., Yu Z. (2024). Superstructure-Assisted Single-Atom Catalysis on Tungsten Carbides for Bifunctional Oxygen Reactions. J. Am. Chem. Soc..

[11] Zheng W., Wang L., Deng F., Giles S.A., Prasad A.K., Advani S.G., Yan Y., Vlachos D.G. (2017). Durable and self-hydrating tungsten carbide-based composite polymer electrolyte membrane fuel cells. Nat. Commun..

[12] Padmakumar M., Dinakaran D. (2021). A review on cryogenic treatment of tungsten carbide (WC-Co) tool material. Mater. Manuf. Processes.

[13] Lan D., Cheng T., Qu Y., Zhang X., Yan X., Suzuki T., Ohishi Y., Wang F. (2022). Tungsten carbide nanoparticles as saturable absorber for Q-switched erbium-doped fiber laser. IEEE Photonics Technol. Lett..

[14] Wang F., Lan D., Zhao J., Qu Y., Zhou X., Zhang X., Cheng T. (2023). Ultrafast fiber lasers at 1560 nm and 1932 nm modulated by WC nanoparticles and d-shaped fibers. Opt. Laser Technol..

[15] Hu B., Shi X.-L., Cao T., Li M., Chen W., Liu W.-D., Lyu W., Tesfamichael T., Chen Z.-G. (2023). Advances in flexible thermoelectric materials and devices fabricated by magnetron sputtering. Small Sci..

[16] Teresi S., Sebe N., Patterson J., Frottier T., Kandazoglou A., Noël P., Sgarro P., Térébénec D., Bernier N., Hippert F. (2023). Spin-Orbit Readout Using Thin Films of Topological Insulator Sb_2_Te_3_ Deposited by Industrial Magnetron Sputtering. Adv. Funct. Mater..

[17] Han X., Zhang H., Jiang S., Zhang C., Li D., Guo Q., Gao J., Man B. (2019). Improved laser damage threshold of In_2_Se_3_ saturable absorber by PVD for high-power mode-locked Er-doped fiber laser. Nanomaterials.

[18] Liu G., Kuang D., Song L., Xu C., Yan C. (2023). Mechanism in damage variation of nanosecond laser-induced damage of germanium sheets in vacuum. Opt. Laser Technol..

[19] Hussain S., Rabani I., Vikraman D., Feroze A., Karuppasamy K., Haq Z.U., Seo Y.-S., Chun S.-H., Kim H.-S., Jung J. (2020). Hybrid design using carbon nanotubes decorated with Mo_2_C and W_2_C nanoparticles for supercapacitors and hydrogen evolution reactions. ACS Sustain. Chem. Eng..

[20] Li Y., Wu X., Zhang H., Zhang J. (2018). Interface designing over WS_2_/W_2_C for enhanced hydrogen evolution catalysis. ACS Appl. Energy Mater..

[21] Krasovskii P.V., Malinovskaya O.S., Samokhin A.V., Blagoveshchenskiy Y.V., Kazakov V.A., Ashmarin A.A. (2015). XPS study of surface chemistry of tungsten carbides nanopowders produced through DC thermal plasma/hydrogen annealing process. Appl. Surf. Sci..

[22] Ferrari A.C., Robertson J. (2000). Interpretation of Raman spectra of disordered and amorphous carbon. Phys. Rev. B.

[23] Ferrari A.C., Robertson J. (2004). Raman spectroscopy of amorphous, nanostructured, diamond–like carbon, and nanodiamond. S.A. Math. Phys. Eng. Sci..

[24] Guo J., Huang D., Zhang Y., Yao H., Wang Y., Zhang F., Wang R., Ge Y., Song Y., Guo Z. (2019). 2D GeP as a novel broadband nonlinear optical material for ultrafast photonics. Laser Photonics Rev..

[25] Gao L., Chen H., Zhang F., Mei S., Zhang Y., Bao W., Ma C., Yin P., Guo J., Jiang X. (2020). Ultrafast relaxation dynamics and nonlinear response of few-layer niobium carbide MXene. Small Methods.

[26] Shang X., Zhang Y., Li T., Zhang H., Zou X., Wageh S., Al-Ghamdi A.A., Zhang H., Si S., Li D. (2024). Nonlinear optical response of niobium telluride and its application for demonstrating pulsed fiber lasers. J. Mater..

[27] Liu J., Yang F., Lu J., Ye S., Guo H., Nie H., Zhang J., He J., Zhang B., Ni Z. (2022). High output mode-locked laser empowered by defect regulation in 2D Bi_2_O_2_Se saturable absorber. Nat. Commun..

[28] Pan H., Chu H., Li Y., Pan Z., Zhao S., Zhao W., Huang W., Li D. (2023). Bismuthene quantum dots integrated D-shaped fiber as saturable absorber for multi-type soliton fiber lasers. J. Mater..

[29] Tinglun X., Xiaoyu W., Zhenni O., Jieling G., Dunlu S., Huaixi C., Xi W., Ke C., Yuzong G. (2024). Peak-power of 25 W passively Q-switched ~2.8 μm Er:YAP bulk laser based on a reflective TaSe_2_ saturable absorber mirror. Opt. Laser Technol..

[30] Feng X.Y., Ding B.Y., Liang W.Y., Zhang L., Liu Q. (2018). MXene Ti_3_C_2_Tx Absorber for a 1.06 μm Passively Q-Switched Ceramic Laser. Laser Phys. Lett..

[31] Su X., Zhang B., Wang Y., Li J., Chen Z. (2018). Broadband Rhenium Disulfide Optical Modulator for Solid-State Lasers. Photonics Res..

[32] Wang J., Xie L., Wang Y., Li H., Zhang X. (2023). High-Damage Vanadium Pentoxide Film Saturable Absorber for Sub-Nanosecond Nd:YAG Lasers. Infrared Phys. Technol..

[33] Wang J., Xie L., Liu J., Li Y., Zhang X. (2025). Nonlinear Optical Response of Thermally Stable Perovskite for Near-Infrared Optical Modulator. J. Mater..

[34] Wang J., Chen Z., Wang Y., Li H., Zhang X. (2020). Molybdenum Disulfide Film Saturable Absorber Based on Sol–Gel Glass and Spin-Coating Used in High-Power Q-Switched Nd:YAG Laser. ACS Appl. Mater. Interfaces.

